# Moving Education Forward, Again!

**DOI:** 10.1371/journal.pcbi.1003390

**Published:** 2013-12-12

**Authors:** Fran Lewitter

**Affiliations:** Bioinformatics and Research Computing, Whitehead Institute, Cambridge, Massachusetts, United States of America

Educating biologists and computational biologists in methods and analyses is an ever-growing challenge. The amount of data and tools available to the scientific community continue to grow, and with that, there is a growing need to teach how to get the most out of this information. The *PLOS Computational Biology* Education section was launched in January 2006 [1] to address this challenge, with the first contribution published in April of that year [2]. In these past eight years, we have published more than 50 Education articles on different topics and have started two specialized Education collections: “Bioinformatics: Starting Early” [3], which addresses the needs of teaching bioinformatics at the secondary school level, and the first PLOS online textbook, “Translational Bioinformatics” [4].

After eight years in the role of editor of this section, I have decided that it is time to step aside to allow fresh ideas to be introduced into building the Education section and new directions to be followed as our science continues to mature. I am delighted that Francis Ouellette (Ontario Institute for Cancer Research, Toronto, Canada) and Joanne Fox (University of British Columbia, Vancouver, Canada) will be the new Education Editors at *PLOS Computational Biology*. They are both leaders in their fields with widespread interests in education and training of various audiences.

It has been extremely fulfilling to be the Education Editor during this time. I have worked with so many talented people on the *PLOS Computational Biology* staff. Special thanks go to Evie Browne and Catherine Nancarrow for helping to get the section off the ground so many years ago. It has also been a pleasure and incredible learning experience to work with Philip E. Bourne, Founding Editor-in-Chief and, more recently, with Ruth Nussinov, Editor-in-Chief.

In addition to the staff (both past and current), it has been wonderful to work with so many creative scientists in the field who have proposed and written articles on diverse topics. And, of course, thanks to all the reviewers who helped improve these articles. In the beginning, I invited people I knew to contribute articles to the Education section. Little by little, as the section matured, proposals were submitted directly to us. I have learned so much reading and editing the published articles and have used this wealth of information to continue to educate and train biologists here at Whitehead Institute. It is also rewarding to hear from colleagues at other institutions about their creative uses of these materials.

I'll end by remarking on the power of networking in the scientific community. While attending the ISMB 2005 conference in Detroit, Michigan, I had a brief conversation with Mark Patterson who was then Director of Publishing at PLOS. I had worked with him on some of his earlier ventures and chatted with him about some of my then current interests. I told him that I had been focusing on training and educating biologists in computational methods. My group (Bioinformatics and Research Computing) at Whitehead Institute had developed numerous three-day courses to teach biologists at the Institute and in the greater Boston community. He said something to the effect, “You should talk to Phil Bourne who recently became Editor-in-Chief of a new journal, *PLOS Computational Biology*. He is interested in something related to education for the journal.” It was that five- or ten-minute conversation that led to a very rewarding activity and rich experience during these past eight years. My advice: don't be shy; share your thoughts and ideas with others and see where it leads. For me it was an extremely short but valuable conversation.

I look forward to following *PLOS Computational Biology* and the Education section as they continue to grow in ways unimaginable eight years ago.
